# Cyclic GMP and Cilia Motility

**DOI:** 10.3390/cells4030315

**Published:** 2015-07-31

**Authors:** Todd A. Wyatt

**Affiliations:** 1 VA Nebraska-Western Iowa Health Care System, Research Service, Department of Veterans Affairs Medical Center, 4101 Woolworth Avenue, Omaha, NE 68105, USA; 2 Department of Environmental, Agricultural, and Occupational Health, College of Public Health, University of Nebraska Medical Center, Omaha, NE 68198-5910, USA; E-Mail: twyatt@unmc.edu; Tel.: +1-402-559-3817; Fax: +1-402-559-8210; 3 Pulmonary, Critical Care, Sleep & Allergy Division, Department of Internal Medicine, 985910 Nebraska Medical Center, Omaha, NE 68198-5910, USA

**Keywords:** cilia, cGMP, signal transduction, history, review

## Abstract

Motile cilia of the lungs respond to environmental challenges by increasing their ciliary beat frequency in order to enhance mucociliary clearance as a fundamental tenant of innate defense. One important second messenger in transducing the regulable nature of motile cilia is cyclic guanosine 3′,5′-monophosphate (cGMP). In this review, the history of cGMP action is presented and a survey of the existing data addressing cGMP action in ciliary motility is presented. Nitric oxide (NO)-mediated regulation of cGMP in ciliated cells is presented in the context of alcohol-induced cilia function and dysfunction.

## 1. Introduction

The motile cilia, as opposed to the primary cilia, serve numerous functions that are predicated upon the movement of the cilia. Single cell organisms depend upon the cilia to provide locomotion and feeding. Cilia also provide a chemosensory role for some organisms. Olfactory cilia motility is essential for the transduction of the sense of smell. Ependymal cilia play an essential role in brain development. The cilia lining the oviduct facilitate movement of the ovum. With regard to human disease, the most studied aspect of cilia movement is the role of lung mucociliary clearance as a cornerstone of innate protection from environmental inhalation injury. 

Mammalian systems commonly use cyclic guanosine 3′,5′-monophosphate (cGMP) for the purposes of movement. For example, cGMP enhances neutrophil chemotactic migration [1], dictyostelium locomotion [2], neuronal cell migration and neurite extension [3], and smooth muscle cell relaxation [4]. Even plants may utilize cGMP systems in movement as stomatal closure is regulated in a nitric oxide-cGMP dependent manner [5]. In this context, it is well established that cGMP is an important regulator of motile cilia movement.

## 2. History of cGMP 

**Figure 1:**
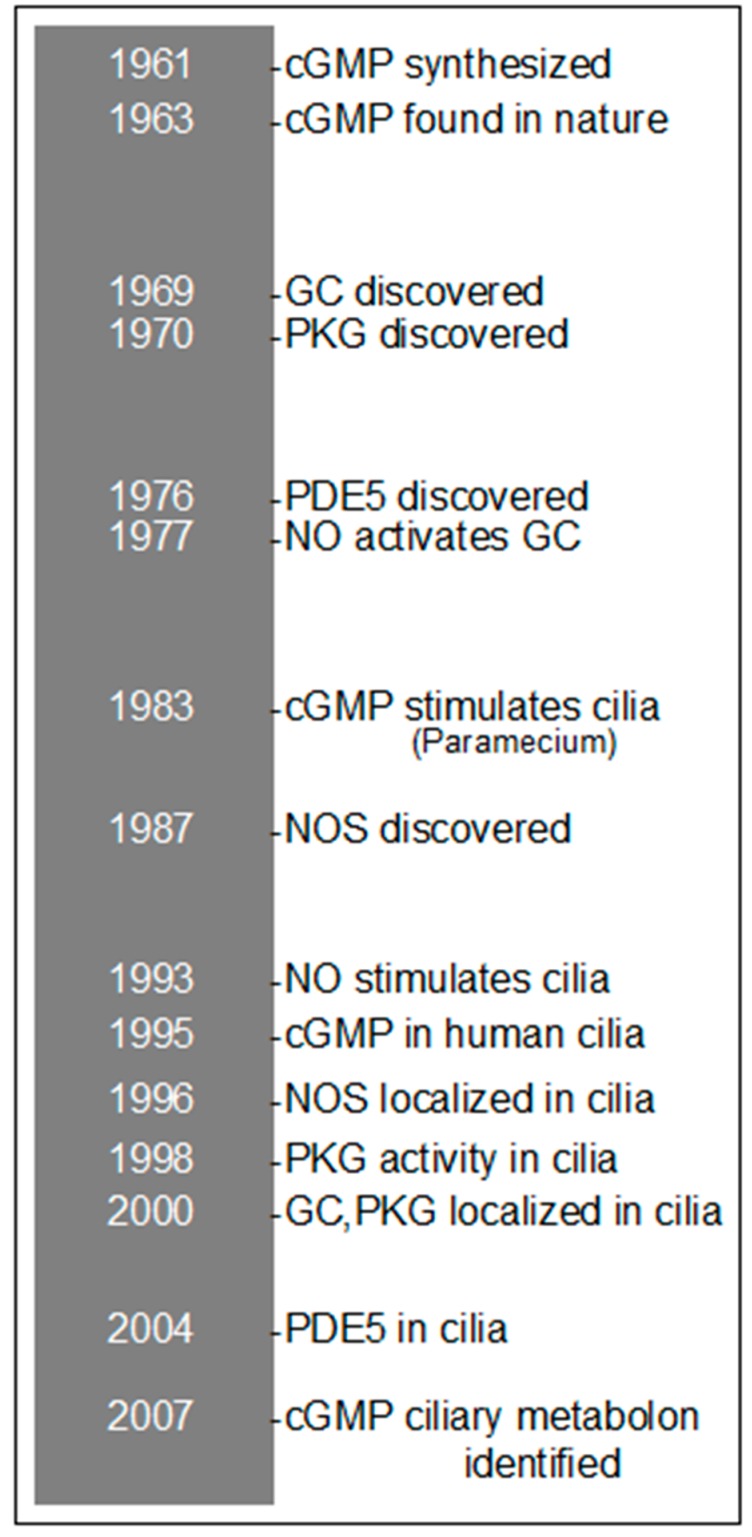
50-year timeline of cGMP and cilia research.

First synthesized in 1961 by Michael Smith in the laboratory of Ghobind Khorana [6], cGMP was subsequently identified in nature by Price et al. in 1963 [7] (see Figure 1). Discoveries of hormone stimulation of cGMP [8] and the enzyme catalyzing the conversion of guanosine-5′-triphosphate (GTP) to cGMP, or guanylyl cyclase (GC) [9], were made in the laboratory of Earl Sutherland, whose earlier discovery of cyclic adenosine 3′,5′-monophosphate (cAMP) [10] was the antecedent to these advancements in the field of cGMP. In contrast to cAMP, whose primary cellular receptor is cyclic AMP-dependent protein kinase (PKA), cGMP has several binding targets. These consist of cyclic GMP-dependent protein kinase (PKG), cGMP-binding, cGMP specific phosphodiesterase (PDE5), cGMP-binding cation channels, and in high concentrations, PKA (Reviewed in [11]). Being short-lived, nitric oxide (NO) must rapidly bind to its target, soluble GC. Otherwise, NO meets a fate of producing reactive nitrogen or oxygen radicals, or the adenosine diphosphate (ADP)-ribosylation of proteins. Cyclic GMP can also be synthesized in an NO-independent manner through the action of particulate GCs activated through the binding of specific target peptides.

A primary receptor for cGMP action, PKG was first discovered in the laboratory of Paul Greengard in 1970 [12]. Localized in both the cytoplasm and membrane of cells, levels of PKG are significantly high in the lungs [13] with a specific concentration compartmentalized to the apical surface and cilia of airway epithelium [14].

While cGMP forms via GC activation, cGMP is converted to the biologically inert 5′-GMP through the action of cGMP-phosphodiesterase (PDE). Several different cGMP-PDEs have been identified, of these are the cGMP-stimulated PDE, the retinal rod outer segment PDE, a cGMP-stimulated PDE found in Dictyostelium, and PDE5 (Reviewed in [15]). PDE5 was first identified in the lungs by Lincoln and Corbin [16] as a cGMP-binding protein unique from PKG. Indeed, PDE5 has been identified in ciliated airway epithelium [17], along with PDE2 [18] and PDE4 [19]. These PDEs function to hydrolyze respective cyclic nucleotides, thus returning cilia beating to homeostatic baseline levels.

Thus, one mechanism for stimulated increases in cilia beat results from elevations in intracellular NO due to the activation of nitric oxide synthase (NOS) resulting in the stimulation of a soluble GC. The subsequent conversion of GTP to cGMP via this cyclase action binds to apical cell and cilia-associated PKG. Axonemal phosphoprotein targets for PKG putatively lead to an increase in dynein ATPase activity. The hydrolysis of adenosine triphosphate (ATP) by the dynein ATPase provides the energy required for microtubule sliding. Unidirectional sliding of outer doublets of microtubules produced by the ATP-powered dynein arms generates ciliary movement. The current dogma of cGMP action has been applied to the regulation of cilia motility and investigated using numerous models of ciliated cells and tissues.

## 3. Model Systems for Studying cGMP Regulation of Cilia

### 3.1. Photoreceptor 

Prior to studies linking cGMP regulation of motility, cGMP action was characterized in non-motile cilia systems. Light-stimulated excitation causes a decrease of the cGMP concentration in vertebrate photoreceptor cells. The relatively high amount of GC in the inner segments and the cilia may contribute—at least in part—to the actual concentration and the time course of concentration changes of the cGMP concentration in rod outer segments [20]. Photoisomerization of rhodopsin activates a heterotrimeric G-protein cascade leading to closure of cGMP-gated channels and hyperpolarization of photoreceptor cells [21]. C-type natriuretic peptide (CNP) significantly elevates cGMP levels in ocular nonpigmented ciliary epithelial cells [22]. Even though non-microvillar photoreceptor cilia have distinctly different regulatory features than those of microvillar photoreceptors, invertebrate ciliary photoreceptors also regulate light adaptation through cGMP and PKG [23]. The elevation of cGMP levels decreases light sensitivity, as cGMP analogs are capable of potentiating light-induced desensitization.

### 3.2. Olfactory Receptor Cilia

This conductance resembles the cyclic GMP-gated conductance that mediates phototransduction in rod and cone outer segments, but differs in that it is activated by both cAMP and cGMP. Odorant-stimulated increases in cyclic nucleotide concentration lead to an increase in membrane conductance depolarization [24]. Subsequent studies have shown a greater affinity for cAMP by this channel [25]. The olfactory cilia of rats contain high levels of soluble GC and can be stimulated by odorants to generate large concentrations of cGMP [26]. Cyclic GMP is equally effective at activating cAMP-gated channels in olfactory receptor cells of toads [27]. In rats, the responsiveness of olfactory sensory cells is governed by a cytosolic type-I PKG [28]. PDE2 and GC are both significantly expressed in olfactory cilia where odorant signaling is initiated [18]. Odorant activation of GC and cGMP production stimulates PDE2 to hydrolyze both cAMP and cGMP to regulate cyclic nucleotide gated channel control of neural axon signaling. Low levels of cGMP are sufficient to stimulate sustained calcium flux signaling through the cyclic nucleotide gated channels in salamander ciliated olfactory cells [29]. 

Cyclic GMP may be an important mechanism for odorant “imprinting” as well. Coho salmon pre-exposed to an alcohol (phenylethyl alcohol) produced a significantly higher cGMP response after a two-month re-exposure to the alcohol as compared to naïve salmon [30]. No such sensitization occurred with adenylyl cyclase (AC). Interestingly, in rat primary nasal cilia where GC and PKG exclusively localize in the tissue, odor-induced cGMP elevations augmented the cAMP-pathway signaling in a PKG-dependent manner [31]. In fact, PKG was just as effective as forskolin at activating rat olfactory AC. This is not unlike the observed alcohol-mediated regulation of PKA, which requires the activation of PKG [32]. In rat olfactory neurons, a link between cAMP and NOS was demonstrated to involve the rise in cytosolic calcium concentration elicited by either plasma membrane calcium channel activation or calcium mobilization from stores via the guanine nucleotide exchange factor Epac [33].

Primordial chemosensation can be thought of as a type of olfactory response. In Caenorhabditis elegans, chemosensation to locate food uses cGMP to open cGMP-gated channels in response to G-protein signaling [34]. When a cGMP-PDE is activated, the cGMP-gated channel closes, but when a GC is activated, the cGMP-gated channel opens allowing for sodium or calcium-induced depolarization. Such studies implicating cGMP regulation of cilia photoreceptor action and olfactory chemosensation underlaid the search for a cGMP role in motile cilia.

## 4. cGMP Regulation of Protozoan Cilia

### 4.1. Paramecium

In Paramecium, swimming direction is controlled through voltage-gated calcium channels located in the membrane of the cilia. Depolarizing stimuli elevate calcium leading to a reversal of the cilia power stroke and resulting in backward movement.

While cGMP contributes to an equal stimulation of forward swimming as cAMP, swimming direction is shifted by cGMP from a right to a left-handed helical path resembling backward swimming in the presence of calcium [35]. A rapid (10 min) stimulation of Paramecia with 2% ethanol resulted in a 123% increase in forward swimming speed [36]. Larger concentrations (5%) of ethanol desensitized the cell and decreased swimming velocity. Alcohol stimulated greater increases in cGMP in the ciliated than the deciliated cells. High alcohol (5%) stimulated large amounts of cGMP formation within 30 s, which very rapidly returned to baseline levels by 2 min, as a membrane calmodulin-dependent GC stimulates cGMP levels [37]. A similar dark-adapted protozoan, *Stentor coeruleus*, demonstrates a light-aversion response whereby the reverse or backup motility in response to light is governed by a cGMP-stimulation of cilia [38]. 

Cyclic GMP was originally shown to bind to an 85 kDa protein, likely corresponding to PKG [39]. Subsequent work showed that the 77 kDa cGMP-kinase found in Paramecium is a primordial PKG that exists in monomeric, not the dimeric form as found in mammals [40]. This PKG was found to both bind cGMP and be autophosphorylated by it. In addition, Paramecium was found to contain PKG at a high specific activity relative to other organisms. In early work, Alton Steiner demonstrated that co-localization of cGMP, PKG, and GC were observed by immunocytochemistry in Paramecium to be localized on the cilia with little cGMP machinery staining in the cell body or nucleus [41]. Subsequently, multiple PKGs have now been identified in Paramecium of variable structure, some resembling that of higher eukaryotes [42]. Regardless of the isoenzyme, PKG is always localized to the cilia with the most intense PKG staining found at the oral cavity [42]. It is interesting to note that such a concentration of PKG at the oral cavity functionally correlates with that region of the cilia demonstrating the highest values for cilia beat frequency (CBF; [43]).

Cyclic GMP stimulation of PKG results in the endogenous phosphorylation of several cilia-associated proteins in Paramecium [39]. Specifically, phosphoproteins contained in the 22 S dynein heavy chains of 160 kDa and 30 kDa are preferentially phosphorylated by cGMP [44]. The specificity of Paramecium cilia substrates (a 29 kDa protein substrate) for a unique cyclic nucleotide kinase was demonstrated by showing a lack of 22 S dynein phosphorylation by purified bovine catalytic subunit (C-subunit) [45]. Cyclic GMP stimulated *in vitro* phosphorylation of several proteins in isolated cilia [46].

Using a cortical sheet model of Paramecia cilia covered with intact membrane, cyclic nucleotides were demonstrated to exert their regulatory effects without a membrane potential and increase CBF [47]. Stimulation of isolated ciliary axonemes by cAMP led to the phosphorylation of 29 kDa and 65 kDa proteins and cGMP caused the phosphorylation of a 42-kDa protein. In an effort to describe the seemingly different observations of cAMP and cGMP action on CBF in Paramecium, a mathematical model for calcium-dependent cyclic nucleotide metabolism was employed [48]. In this model, changes in calcium concentration can lead to increases or decreases in CBF at specific concentration ranges. Furthermore, this model suggests that cAMP and cGMP operate in combination, as opposed to regulating opposite beating. Such a model would agree with cyclic nucleotide regulation of mammalian ciliated cells.

### 4.2. Tetrahymena 

Tetrahymena metabolize calcium during cilia regeneration. There is a net increase in calcium uptake prior to the reinitiating of motility. The increase coincides with an increase in the intracellular level of cGMP [49]. Insulin was shown to stimulate rapid (30 s–1 min) cGMP localization to the cilia of Tetrahymena [50].

## 5. Other Models of cGMP and Cilia Action

A limited number of other studies examining cGMP and cilia have been reported in snails and sea urchin larva. Cilia drive rotation of the embryo in the egg capsule of *Helisoma trivolvis* to promote oxygen diffusion. In these embryos, it was shown that cGMP stimulates CBF in a NO- and GC-dependent manner [51]. While NO and cGMP activity were demonstrated to be essential for stimulated CBF, serotonin only increased CBF in a subset of embryos, suggesting an additional pathway of CBF stimulation. The role of cGMP was also examined in sea urchin larva [52]. 

### 5.1. Animal Models of cGMP-Mediated Cilia Motility

#### 5.1.1. Frog

Using frog palate motile cilia, an interactive relationship was identified for acetylcholine-stimulation of CBF [53]. In this study, cholinergic stimulation of cilia was initiated by elevated calcium then followed by PKG action shortly before the action of PKA. The action of calcium-calmodulin is hypothesized to regulate the dual pathway stimulation of both cyclases, with cGMP/PKG action required for PKA-mediated effects and PKA capable of producing a feedback regulation of cGMP levels [53]. Interestingly, this acetylcholine-mediated dependence of PKA-stimulated CBF on upstream PKG action is also observed in the mammalian ciliary response to ethanol [32].

#### 5.1.2. Rat

The effects of cGMP on cilia motility were first characterized in cultured rat tracheal epithelial cells by Roger Johns et al. [54,55]. In this study, elevated levels of NO could be localized preferentially to the ciliated cells and CBF modulated via the regulation of NO levels utilizing both donors and inhibitors. NO-mediated regulation of CBF was furthermore shown to require the downstream action of GC and PKG. However, PKG activity was not directly measured, but a non-specific inhibitor (KT5823) demonstrated the ability to block L-arginine enhanced CBF at concentrations much higher than the putative Ki for PKG. Interestingly, such a high inhibitor concentration may suggest the importance of PKA in also mediating CBF, as KT5823 has previously been shown to not be an effective inhibitor of PKG [56]. Importantly, this report represents the first utilization of a PDE5-specific inhibitor to enhance CBF.

#### 5.1.3. Rabbit

Inexplicably, regulation of cilia by cGMP is reversed in rabbit studies. First noted by Tamaoki *et al.*, the atrial natriuretic factor-stimulated elevation of cGMP levels in rabbit tracheal ciliated epithelium led to a significant slowing of the cilia. This was re-enforced through the use of both GC and cGMP-PDE inhibitors [57]. Subsequently, it was reported that intracellular calcium levels must be elevated in the rabbit ciliated airway epithelium before cGMP stimulation of CBF could be achieved [58]. It was furthermore demonstrated that two phases of cGMP-mediated stimulation of CBF occur in rabbit: A Calcium-independent rapid stimulation phase that is PKG-dependent and a transient calcium-dependent subsequent stimulation that is PKG-independent [59]. 

#### 5.1.4. Porcine

Western blots of porcine cilia axonemes probed for PKG I-alpha demonstrated the presence of PKG on the ciliary axoneme [60]. Numerous cilia substrates were also identified to be phosphorylation targets of PKG. In intact porcine tissue, cGMP stimulated cilia substrate phosphorylation of distinct proteins 27, 37, and 44 kDa in size.

#### 5.1.5. Bovine

The first direct catalytic measurement of PKG (and PKA) activity in ciliated cells was measured in bovine bronchial epithelial cells stimulated with sodium nitroprusside (SNP) or cGMP analogs (or beta agonists or cAMP in the case of PKA) [61]. This established a parallel action for cyclic nucleotides in the regulation of cilia beating in mammals. This parallel pathway regulation was further expanded to include the identification of PDE activities in bovine ciliated bronchial epithelium specific for cAMP (PDE4; [19]) and cGMP (PDE5; [17]). Ethanol represents such an agent that utilizes both pathways of PKG activation as well as PKA in order to produce a rapid stimulatory response [32]. Furthermore, lower concentrations of combined cAMP and cGMP were as effective at stimulating bovine bronchial cilia beat as higher concentrations of either nucleotide alone. Because cyclic nucleotide cross-activation was not seen at such low concentrations, this suggests that a dynamic cooperativity of kinase action can occur. Functional PKG catalytic activity was localized to the isolated bovine axoneme [62]. The ATP-mediated CBF of detergent extracted bovine ciliary axonemes could be further stimulated by cGMP. This cGMP-mediated stimulation of axonemal “bending” was inhibited by the antagonist analog (Rp-cGMPS) of cGMP. As with intact ciliated cells, maximal axoneme stimulation was accomplished by the combination of cGMP and cAMP, further suggesting PKG-PKA cooperativity. Indeed, a functional cyclic nucleotide metabolon was identified in the ciliated bovine bronchial epithelial cell at the apical membrane, which included co-localized NOS3 (endothelial NOS; eNOS), GC-beta, PKG-I and PKG-II. These proteins were all closely associated at an A-kinase anchoring protein (AKAP) targeting protein with the cAMP-associated machinery of AC and PKA [14]. The intact ciliary metabolon on isolated axoneme organelle preps was demonstrated to directly respond to rapid stimulation by alcohol to elevate NO and activate a soluble AC, leading to the sequential activation of PKG followed by PKA [63]. In this manner, alcohol rapidly and transiently stimulated axonemal CBF through the combination of both PKG and PKA.

#### 5.1.6. Human

C-type natriuretic peptide (CNP) stimulated increased CBF in human polarized ciliated epithelium in culture through the elevation of intracellular cGMP levels [64]. Under the conditions used, CNP did not elevate cAMP levels. Interestingly, in this study, SNP failed to stimulate soluble GC and increase CBF, even though in previous work [64] these same investigators showed that SNP stimulated cGMP in airway epithelium. Using human nasal epithelial tissue, modulation of NO by l-arginine and NOS inhibitors altered methacholine stimulation of CBF. Similarly, a cyclooxygenase inhibitor and a PKG inhibitor blocked methacholine, but not cAMP-mediated, stimulation of CBF, thus establishing a dual pathway activation of mammalian CBF that also involved cGMP as well as cAMP [65]. Methacholine-stimulated increases in adenoid tissue CBF was enhanced by l-arginine and inhibited by a NOS inhibitor, l-NG-Nitroarginine Methyl Ester (L-NAME), thus implicating a role for NO in the methacholine response [66].

#### 5.1.7. Mouse

In addition to studies of photoreceptor and olfactory function that have been conducted in mouse, regulation of murine cilia by cGMP has been reported in both nasal and tracheal epithelium [67]. Using a mouse model of CBF, Jiao et al. confirmed the cilia stimulatory effects of activated NOS [68], GC [32], and PKG [61]. Similar to previous immunolocalization studies performed in rat [69] and bovine [14] ciliated epithelium, NOS3, soluble GC beta, and PKG were also shown by histology to be present in both nasal and tracheal epithelium [67]. A role for NOS3 in the stimulation of mouse cilia has been subsequently demonstrated with regard to regulation by heat shock protein 90 [70], antioxidants [71], and asymmetric dimethylarginine [72]. Because mice deficient in all 3 isoforms of NOS can be generated [73], controlled *in vivo* exposure studies of cilia with regard to NO action are likely to be advanced in the mouse model.

## 6. Nitric Oxide and Cilia

While olfactory cilia were the first to be shown to have a soluble GC-sensitive cGMP response, which could be blocked by NOS inhibitors [26], the first evidence of such NOS action on airway cilia was reported by Sisson *et al*. [68,74]. Similar findings of cilia beating stimulatory regulation by NO were subsequently confirmed by others [58,75,76]. While cGMP was established as the downstream product of NO action, it was clearly established that cAMP-mediated stimulation of the cilia represented a distinct pathway as well [65,77]. In these studies, direct cilia stimulation with cGMP analogs was not blocked by NOS inhibitors. Congruent with these observations, cilia stimulatory agents, such as acute ethanol, which stimulate NO can be blocked with NOS inhibitors [78]. Ciliated tracheal epithelial cells were found to contain NOS3, soluble GC, and PKG I-beta by immunohistochemical localization [69]. These key NO-associated regulators were subsequently identified on the ciliary metabolon located at the apical surface of the cell at the base of the cilia [14]. It has been shown that specific subcellular localization of cGMP synthesis can exist, allowing for differential regulation of cGMP production by natriuretic peptides and NO [79]. Thus, the distinct apical and axonemal localizations of NOS and PKG in ciliated cells [14] may likely govern distinct stimuli responses.

NOS2 (inducible NOS; iNOS) was localized by immunofluorescence microscopy in lung epithelium [80]. In a study of nasal epithelium, tumor necrosis factor alpha increased NOS2 expression, but exhibited contrasting concentration-dependent effects on CBF [81]. Histologic and electron microscopic localization for NOS2 is decreased in the nasal sinuses of individuals with sinusitis and sepsis [82]. This decrease in NOS2 was correlated with decreased exhaled NO levels. Likewise, a trend (not significant when all outliers were included) toward lower NOS2 was reported in the nasal samples of patients with primary ciliary dyskinesia, but no such changes in NOS3 were detected [83]. Thus, the role of NOS2 in nasal epithelium may be different from that of NOS3 in the lung epithelium, although the impact of inflammation and source of exhaled NO may serve as complicating factors. Lastly, NOS1 (neuronal NOS; nNOS) was also localized to the ciliary axoneme using immunofluorescence antibody staining [84]. Because all three NOS isoforms seem to be present in ciliated epithelium, it is possible that differential regulation of NO production could be orchestrated by localized NOS isoforms even though the net effect would be cyclase-driven production of cGMP. 

## 7. Alcohol and the NO-cGMP Ciliary Response

**Figure 2:**
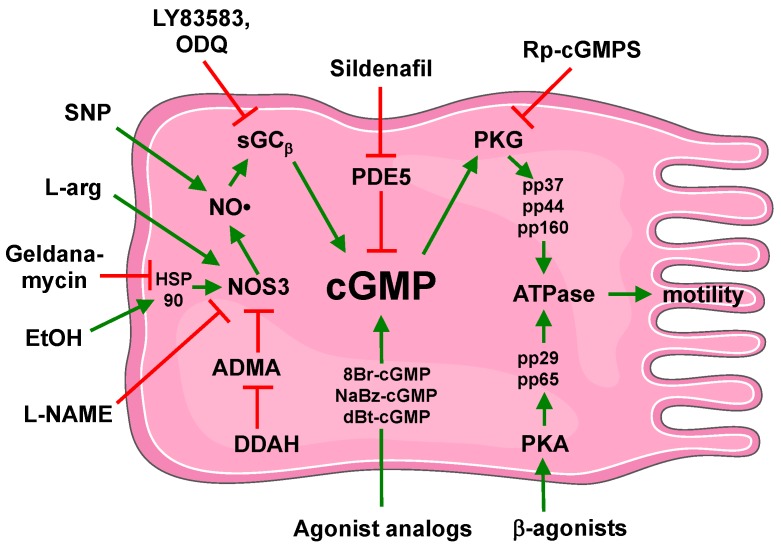
Schematic of cGMP action in cilia motility. Stimulation pathways indicated by green arrow-headed lines. Inhibition pathways indicated by red bar-headed lines. Adapted and modified from Servier Medical Art image bank through Creative Commons Attribution 3.0 Unported.

As previously stated, ethanol rapidly stimulated forward swimming speed, while higher concentrations of alcohol for longer periods of time decreased swimming speed in Paramecium [36]. Likewise, mammalian cilia can be rapidly stimulated by alcohol, yet in time become desensitized to further stimulation after a sustained exposure to alcohol. Furthermore, individuals with alcohol use disorders have long since been clinically observed to be at higher risk for lung infections (reviewed in [85]). This unique effect of ethanol on cilia has served as an important model system for the elucidation of NO-mediated cGMP action on the regulation of cilia beating (see Figure 2). 

Alcohol is unique to human beings as it is a substance tolerated well in millimolar concentrations without lasting toxicity. Blood alcohol levels can range from a couple of drinks within an hour (~10 mM) to an extreme survival case report of 330 mM [86]. Rapid *in vitro* exposure of 20–100 mM ethanol to bronchial epithelial cells results in the stimulation of CBF for 1–2 h [74]. This same alcohol stimulatory effect was observed with *in vivo* alcohol feeding of rats [87], mice [88], and sheep [89] for up to a week. Alcohol stimulates NOS3 in the apical region of the ciliated cell resulting in the production of NO [30]. This is accomplished through the rapid alcohol-stimulated binding of NOS3 to HSP90 on the ciliary axoneme [70]. Similar to dual pathway regulation of cilia as previously discussed in other systems, brief alcohol exposure also stimulates an ethanol-sensitive AC in ciliated epithelial cells which leads to cAMP elevation [32]. However, unique to alcohol, cAMP cannot lead to PKA activation without the ethanol-mediated production of NO [78]. In this regard, the NO-GC-cGMP signal is essential for alcohol-mediated activation of PKA and subsequent cilia stimulation [63]. Approximately 4–6 h after the alcohol-generated elevation of cyclic nucleotide, an increase in PDE4 [19] and PDE5 [17] activities are detected which return kinase activity and CBF levels back to baseline.

In keeping with clinical observations of alcohol abuse and increased pneumonia, chronic ethanol exposure to ciliated cells no longer stimulates CBF, but rather desensitizes the cilia to agents that would otherwise be cilio-stimulatory [90]. Pathophysiologic concentrations of alcohol (50–100 mM) desensitize cilia by 18–24 h *in vitro* and at three weeks *in vivo* [88,91]. This desensitization, termed alcohol-induced ciliary dysfunction (AICD) involves chronic alcohol-mediated effects of oxidants [70], asymmetric dimethylarginine [72], and protein phosphatase 1 [92]. The AICD effect may be associated with the phosphorylation state of the outer dynein arm proteins. For instance, in Chlamydomonas, the phosphorylation state of the outer dynein arm-docking complex (ODA3; DCC1) is altered in the presence of alcohol, and its phosphorylation correlates appears to correlate with AICD [93].

## 8. Conclusion

In summary, cGMP regulation of cilia motility is remarkably conserved across species. Defining the role of cGMP will advance our knowledge of cilia-based functions of olfactory regulation and photosensation in addition to elucidating the molecular nature of motility. While other mediators of cilia also impact motility, defining cGMP action on cilia will be important in understanding innate mucociliary responses to environmental stimuli singularly and in the context of multiple exposures (*i.e.*, alcohol and cigarette smoking).
